# YBX1/YB-1 induces partial EMT and tumourigenicity through secretion of angiogenic factors into the extracellular microenvironment

**DOI:** 10.18632/oncotarget.3764

**Published:** 2015-04-23

**Authors:** Shashi K. Gopal, David W. Greening, Rommel A. Mathias, Hong Ji, Alin Rai, Maoshan Chen, Hong-Jian Zhu, Richard J. Simpson

**Affiliations:** ^1^ Department of Molecular Science, La Trobe Institute for Molecular Science, La Trobe University, Melbourne, Victoria, Australia; ^2^ Department of Surgery, The University of Melbourne, Melbourne, Victoria, Australia

**Keywords:** YBX1, epithelial-mesenchymal transition, EMT, secretome, tumorigenesis

## Abstract

Epithelial-mesenchymal transition (EMT) describes a morphogenetic program which confers mesenchymal cell properties, such as reduced cell-cell contact and increased cell migration and invasion, to epithelial cells. Here we investigate the role of the pleiotropic transcription/splicing factor and RNA-binding protein nuclease-sensitive element-binding protein 1 (YBX1/YB-1) in increasing the oncogenic potential of epithelial MDCK cells. Characterization of MDCK cells expressing YBX1 (MDCK^YBX1^ cells) revealed a partial EMT phenotype, including cytosolic relocalization of E-cadherin, increased cell scattering, and anchorage-independent growth. Subcutaneous injection of parental MDCK cells into NOD/SCID mice did not form tumours. Critically, MDCK^YBX1^ cells established viable tumour xenografts, and immuno-histochemical staining indicated murine vascularization by CD31+ endothelial cells. We analysed the total secretome (containing soluble and extracellular vesicles) of MDCK^YBX1^ cells to investigate regulation of the tumour microenvironment. YBX1 expression elevated release of secreted factors known to enhance angiogenesis (TGF-β, CSF-1, NGF, VGF, ADAM9 and ADAM17), compared to MDCK cells. Importantly, treatment with MDCK^YBX1^ cell-derived secretome increased recipient 2F-2B endothelial cell motility. This defines YBX1 as an oncogenic enhancer that can regulate tumour angiogenesis via release of secreted modulators into the extracellular microenvironment.

## INTRODUCTION

Epithelial-mesenchymal transition (EMT) describes a shift in cellular plasticity whereby epithelial cells lose their phenotype and acquire mesenchymal cell characteristics [1, 2]. A fundamental process during embryogenesis, EMT has also been implicated in stages of tumorigenesis whereby carcinoma cells disseminate from their resident environment and metastasize to secondary sites [3]. The EMT process is considered a multi-stage process involving collaboration of several inducers (HGF [4], PDGF [5], IGF [6], EGF [7]), signalling pathways (TGF-β [8], Wnt [9], Notch [10]), transcription factors (Snail1 [11], Twist [2], ZEB [12]) and cellular mediators (ZO-1 [13], CLDN1 [14], and MUC-1 [15]). Given secreted proteins comprise an assortment of bioactive molecules which can regulate various physiological functions [16, 17], we have been interested in defining the contribution of the extracellular microenvironment during EMT [18–20].

Using the EMT model of MDCK cells and Ras-transformed MDCK (21D1) cells, we have previously performed proteomic-based profiling of the secretome [20, 21], plasma membrane [22], and exosomes [23]. These analyses have revealed several EMT modulators that are differentially expressed, and promote the progression of EMT (i.e., MMP-1 and Wnt-5a). Comparative analysis of exosomes released from MDCK and 21D1 cells revealed 21D1 exosomes contain an EMT signature [23]. This result led us to hypothesize that exosomes may be used as a vehicle to induce EMT in recipient cells upon their uptake [24].

21D1 exosomes uniquely contained the transcription/splicing factor and RNA-binding protein nuclease-sensitive element-binding protein 1 (YBX1/YB-1), and it was the most up-regulated protein compared to MDCK exosomes [23]. YBX1 is a member of the cold shock protein family of proteins, and a master transcription factor regulating an assortment of genes controlling cellular proliferation and development [25, 26], as well as invasion, DNA repair, RNA splicing and exon skipping [27, 28]. Stable expression of YBX1 in MCF10AT (Ras-transformed MCF10A) cells induces EMT via induction of cap-independent translation of mRNAs encoding EMT-promoting factors such as Snail1, and suppression of cap-dependent translation of mRNAs encoding growth-promoting factors [26]. YBX1 has more recently been reported to regulate EMT in lung cancer cells via TGF-β-dependant signalling [29]. Despite these reports, YBX1 has also been reported to play tumour-suppressive roles in cell proliferation and development [30], indicating diverse cellular mechanisms of action. Therefore, we were interested in further investigating the effects of YBX1 in EMT and tumorigenesis.

To test the oncogenic potential of YBX1 we generated MDCK cells stably expressing YBX1 (MDCK^YBX1^ cells). Elevated YBX1 expression in these cells induced the onset of EMT, with MDCK^YBX1^ cells exhibiting cytosolic E-cadherin relocalization, increased expression of Snail1 and Twist, and anchorage-independent growth. Most significantly, MDCK^YBX1^ cells established tumour xenografts *in vivo* in mouse models. The increased tumourigenicity of these cells correlated with elevated secretion of several angiogenic factors in the secretome (containing both soluble and extracellular vesicle components). Furthermore, addition of MDCK^YBX1^ secretome to endothelial cells elevated recipient cell migration, compared to cells stimulated with MDCK. We report YBX1 as an oncogenic modulator which enhances EMT progression and angiogenesis through regulation of the tumour microenvironment.

## RESULTS

We have previously shown that stable expression of oncogenic H-Ras in MDCK cells (21D1 cells) induces complete EMT with hallmark features including expression of EMT markers, cell scattering, and enhanced migration and invasion [20–22]. The cellular characteristics which represent both epithelial (MDCK) and mesenchymal (21D1) cells were implemented in this current study as reference points to assess the EMT phenotype when YBX1 is stably expressed in MDCK cells (MDCK^YBX1^).

### Expression of YBX1 induces partial EMT in MDCK cells

YBX1 was overexpressed in MDCK cells, and several clones generated. MDCK^YBX1^ clone 5 (C5) had the highest expression of YBX1 (Supplementary Figure S1a), and subsequently selected for further characterisation.

#### Cell morphology and growth

**Figure 1:**
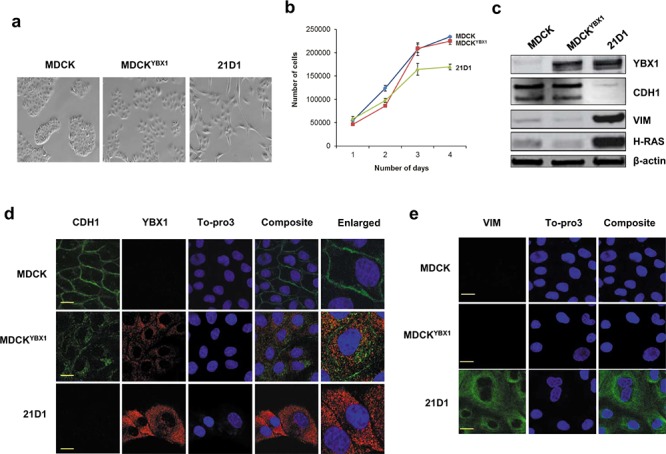
YBX1 overexpression induces partial EMT in MDCK cells **a.** Stable expression of YBX1 in MDCK cells (MDCK^YBX1^) induces cells scattering (10 × magnification) **b.** Cell growth was monitored by counting sub-confluent cell numbers every 24 hr, over 4 days. (*n* = 3; average ± SEM). **c.** Immuno-blot analysis of epithelial (CDH1), mesenchymal (VIM), and expression of YBX1 and H-Ras. **d.** Confocal microscopy of CDH1 (green), and YBX1 (red) expression (scale bar = 10 μm). **e.** Confocal images of cytoskeletal VIM (green) (scale bar = 10 μm).

MDCK^YBX1^ cells still retain a “cobble-stone-like” appearance, but have slightly increased scattering compared to MDCK cells (Figure 1a). The growth rate of MDCK and MDCK^YBX1^ cells is not significantly different (Figure 1b).

#### Expression of EMT markers

As expected, MDCK^YBX1^ cells have elevated levels of YBX1 compared to MDCK cells (Figure 1c), and YBX1 exhibits cytosolic distribution (Figure 1d). Expression of YBX1 in MDCK cells did not increase the expression of mesenchymal marker vimentin, compared to MDCK cells (Figure 1c and 1e). Similarly, overall expression of epithelial marker E-cadherin (CDH1) was not reduced in MDCK^YBX1^ cells (Figure 1c). However, compared to the plasma membrane/cell junction distribution of CDH1 in MDCK cells, CDH1 appears to be internalised in MDCK^YBX1^ cells, with increased cytosolic localization (Figure 1d). Examination of nuclear cell extracts showed modest elevation of EMT transcription factors Snail and Twist in MDCK^YBX1^ cells, relative to extracts from MDCK cells (Supplementary Figure S1b–S1c).

#### Wound healing, cell migration and invasion

**Figure 2:**
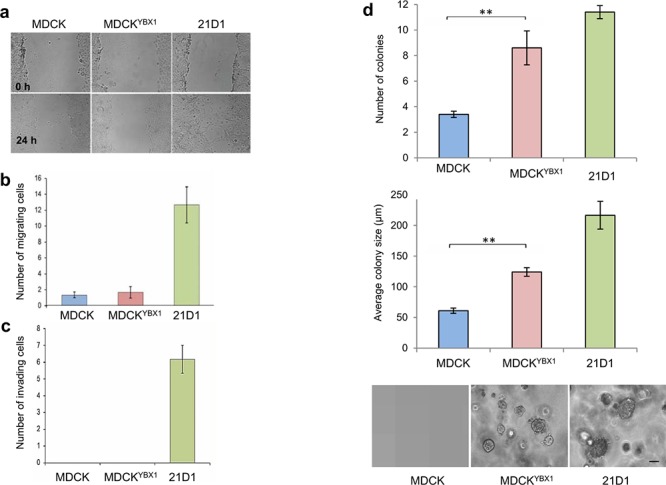
YBX1 facilitates anchorage-independent growth *in vitro* **a.** Cell migration over 24 hr assessed by wound-healing assays and phase-contrast microscopy (4 × magnification). **b.** Cell migration as assessed by transwell assay using 8.0 μm membrane culture inserts. 24 h after cell seeding, migrating cells that passed through the transwell were stained with DAPI and counted (*n* = 3; average ± SEM). **c.** Transwell invasion assays were conducted using 8.0 μm membrane inserts coated with 1 mg/mL Matrigel. 24 h after cell seeding, invading cells were stained with DAPI and counted (*n* = 3; average ± SEM). **d.** Colony forming assays were performed in soft agar using CytoSelect 96-well cell transformation kit. 2,500 cells were inoculated and cultured for 7 days. Number of colonies were manually counted from 5 random fields of view (for each replicate) and colony size measured and quantified. Images are representative. (scale bar: 100 μm) (*n* = 3; average ± SEM; **P < 0.01).

Wound healing assays and transwell assays were employed to assess cell migration, and show that MDCK and MDCK^YBX1^ cells have similar migration ability (Figure 2a–2b). Similarly, assessment of cell invasion showed no change between the cell lines (Figure 2c).

#### Anchorage independent growth *in vitro*

Compared to MDCK cells, a significantly elevated total number of MDCK^YBX1^ cell colonies were quantified in the colony formation assay. (Figure 2d). Additionally, the average size of each colony was also increased in the soft agar, indicating that YBX1 enhances cell transformation (Figure 2d).

Overall, using the 21D1 cell phenotype as an indicator for ‘complete EMT’, expression of YBX1 in MDCK cells induced the onset of some EMT features, e.g., cytosolic localization of CDH1, and increased expression of EMT transcription factors Snail and Twist in MDCK^YBX1^ cells. A striking feature was the observation that in soft agar MDCK^YBX1^ cells were able to form colonies and proliferate independently of substratum attachment.

### MDCK^YBX1^ cells establish subcutaneous tumour xenografts

**Figure 3:**
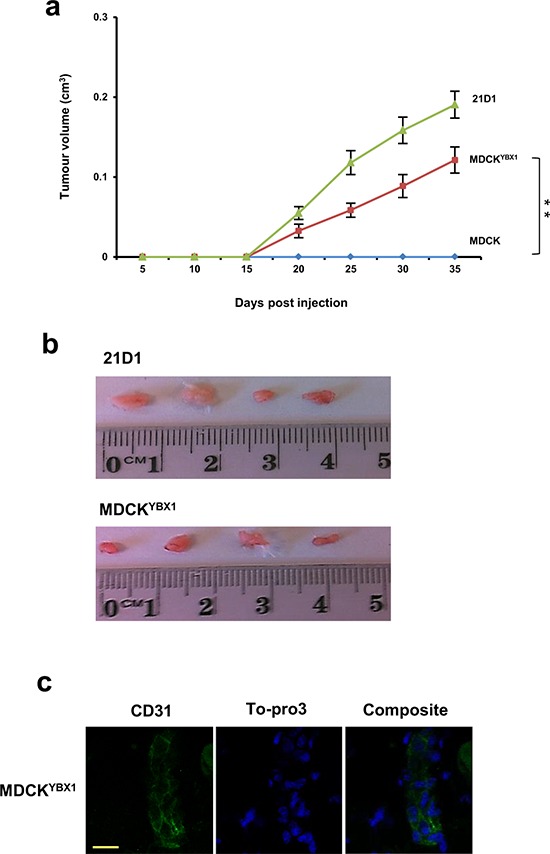
MDCKYBX1 cells generate tumour xenografts **a.** 1 × 10^6^ cells/site were injected subcutaneously into NOD/SCID mice, in both inguinal regions. Tumour volumes were measured at indicated times. (*n* = 8; average tumour volume ± SEM; **P < 0.01). **b.** Tumours were excised after 5 weeks, and representative images shown. **c.** Immunohistochemistry of CD31 (green) expression in MDCK^YBX1^ tumour xenografts (30 μm scale, *n* = 2).

To further explore YBX1 and tumorigenesis, we next subcutaneously injected 1 × 10^6^ MDCK, MDCK^YBX1^, or 21D1 cells into NOD/SCID mice, and monitored tumour growth (Figure 3). MDCK cells did not form a tumour xenograft, while both MDCK^YBX1^ and 21D1 cells established xenografts, and continued to grow for 5 weeks (Figure 3a). After this time, the primary tumour volumes for MDCK^YBX1^ and 21D1 cells were measured to be 0.12 cm^3^ and 0.19 cm^3^, respectively (Figure 3b). Given that MDCK cells do not form a tumour xenograft, this finding demonstrates that increased expression of YBX1 can increase the tumourigenic potential of these cells.

### Identification of MDCK cellular proteins induced by YBX1 overexpression

To further explore underlying proteins that may confer tumourigenic properties to MDCK^YBX1^ cells, we used proteomics to identify MDCK proteins differentially expressed as a consequence of YBX1 overexpression. As MDCK cells do not form subcutaneous tumours in NOD/SCID mice, we analysed the cellular lysates from *in vitro* tissue cultured cells, and identified 925 and 830 (consistent in both replicates) proteins expressed in parental MDCK and MDCK^YBX1^ cells, respectively (Supplementary Figure S2, Supplementary Table S1). Of these, 57 proteins were deemed to be significantly up-regulated (fold-change > 2 and *p*-value < 0.05) in MDCK^YBX1^ cells, relative to MDCK cells (Supplementary Table S2). These enriched proteins in MDCK^YBX1^ cells included ANXA7, RPLP2 and SERPINC1. Interestingly, several of these were mitochondrial constituents, including SLC25A12, CAV2, SDHB, DHRS2, ACADSB, PNPT1, PITRM1, TIMM50, SLC25A1, IARS2, and OAT, and we hypothesize that these mitochondrial proteins may contribute to the metabolic requirements needed for tumour formation. In addition, proteins implicated in inhibiting anchorage-independent growth, (DSG2 and BAX) were decreased in MDCK^YBX1^ cells in comparison to MDCK (Supplementary Table S1).

### Analysis of MDCK^YBX1^ tumour xenografts

Proteomic analysis of MDCK^YBX1^ tumour xenografts revealed 428 proteins identified in MDCK^YBX1^ tumour xenografts (Supplementary Figure S3, Supplementary Table S3), and EMT markers (VIM, FN1 and S100A4) and proteins involved in cancer progression (H-Ras and CLU) were identified. Moreover, we were able to validate the expression of 16/57 differentially expressed proteins in MDCK^YBX1^ tumour xenografts (Supplementary Table S2), some of which were mitochondrial (ACADSB, SLC25A1, IARS2, and OAT). Interestingly, we observed the abundant expression in the tumours of several proteins known to be involved in angiogenesis, including ANXA2 [31], CA2 [32], ATP5B [33] and HSPB1 [34] (Supplementary Table S3). Examination of tumour sections by immuno-histochemistry revealed expression of CD31, a vascular endothelial marker consistent with significant vascularisation (Figure 3c).

### MDCK^YBX1^ cells secrete factors which enhance endothelial cell migration

**Figure 4:**
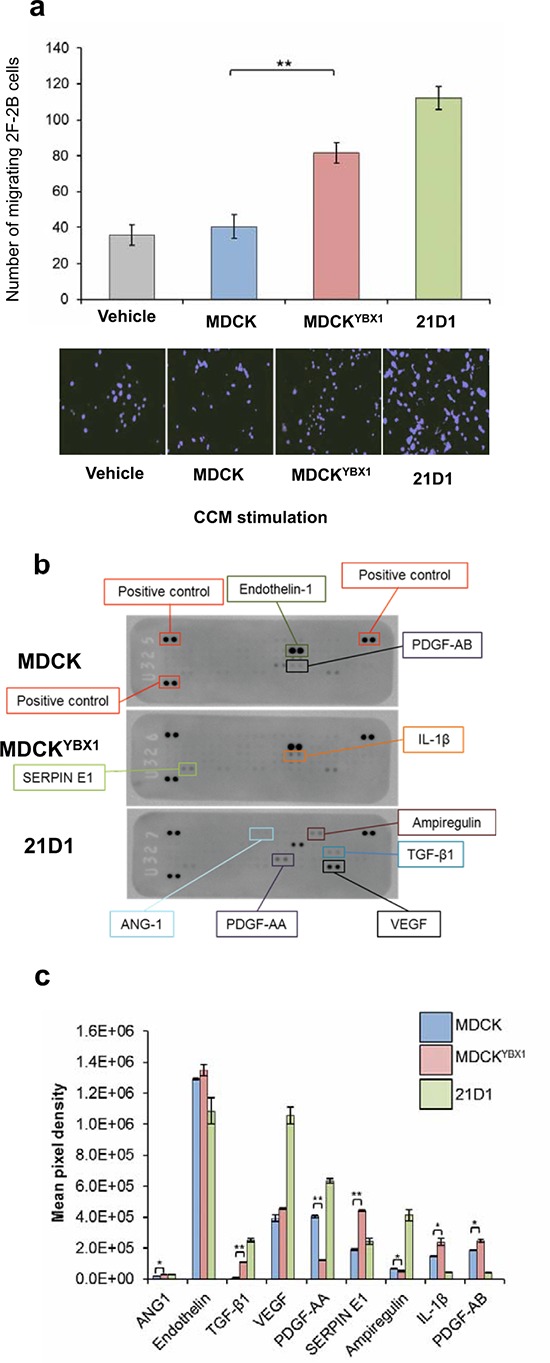
MDCKYBX1 cells secrete angiogenic factors which increase endothelial cell migration **a.** 5 × 10^4^ 2F-2B cells were conditioned with 30 μg of MDCK, MDCK^YBX1^ or 21D1 cell-derived secretome. Cell migration over 24 hr was assessed using Transwell inserts (8.0 μm pore size). Migrating cell nuclei were stained with DAPI, imaged and quantified (*n* = 3; average ± SEM; **P < 0.01). **b.** Cell-derived secretome was profiled using antibody-based angiogenesis arrays. Expression of 55 target proteins in the secretome was assessed. **c.** Relative expression of angiogenic modulators in cell-derived secretome (*n* = 3; average intensity ± STD. *P < 0.05, **P < 0.01).

We hypothesized that elevated YBX1 expression may contribute to tumour angiogenesis, by increasing endothelial cell migration. To test this we collected the secretome (i.e. concentrated conditioned medium containing both soluble secreted proteins and extracellular vesicles) from MDCK, MDCK^YBX1^ and 21D1 cells, supplemented recipient endothelial 2F-2B cells, and monitored cell migration over 24 hr period. Addition of parental MDCK cell secretome did not change 2F-2B cell migration compared to the vehicle (DMEM only) (Figure 4a). However, conditioning with MDCK^YBX1^ secretome promoted 2F-2B motility, with more cells traversing through the transwell (Figure 4a).

To elucidate the molecules responsible for facilitating endothelial cell migration we first used an angiogenesis antibody array to screen the expression of 55 angiogenic-associated factors in the secretome of all three cell lines (Figure 4b). Comparative analysis revealed several cytokines and growth/differentiation factors with increased expression in the secretome from MDCK^YBX1^ cells, compared to MDCK, including SERPIN E1, IL-1β and TGF-β1 (Figure 4c).

### Proteomic characterization of MDCK^YBX1^ cell secretome

With promising differences identified using the angiogenic array, we subjected the secretome from MDCK, MDCK^YBX1^, and 21D1 cells, to in-depth proteomic characterization (Supplementary Figure S4, Supplementary Table S4). Comparison of MDCK and 21D1 secretome identified 111 proteins significantly differentially expressed (fold-change > 2 and *p*-value < 0.05, Supplementary Table S5). This was consistent with our previous studies which reported the up-regulation of extracellular modulators (MMP-1, TIMP-1, SPARC, COL1A2, ITGA6, and ITGA5) in the secretome of 21D1 cells [20–22]. We were interested in examining whether the same molecules were up-regulated in the secretome of MDCK^YBX1^ cells (Supplementary Figure S4b), relative to MDCK, however only 11/111 proteins were common (Supplementary Table S5), indicating a different suite of molecules may drive a more complete oncogenic Ras-induced EMT in 21D1 cells. Comparison of MDCK and MDCK^YBX1^ secretomes (Supplementary Figure S4b), revealed 47 proteins enriched in the secretome of MDCK^YBX1^ cells (fold-change > 2 and *p*-value < 0.05), including many known to be involved in angiogenesis, including proteases (ADAM9, ADAM17), inhibitors (SERPIN C1), and stimulatory/growth factors (CSF-1, VGF, NGF) (Supplementary Table S6). Interestingly, ADAM9, VGF, and CSF-1 were also increased in 21D1 secretome, relative to MDCK, suggesting these molecules may result from increased expression of YBX1 in 21D1 cells (Figure 1c).

## DISCUSSION

Unlike 21D1 cells (H-Ras-transformed MDCK cells) which exhibit a full EMT phenotype [20–22], stable expression of YBX1 in MDCK cells induced partial EMT characteristics, including relocalization of E-cadherin, greater cell scattering and anchorage-independent growth. The relocalisation of E-cadherin away from the plasma membrane is known to be associated with the early stages of EMT, and has been previously reported in NBT-II cells undergoing IGF-II-stimulated EMT [35]. Additionally, we detected modest increases in Snail1 and Twist expression in MDCK^YBX1^ cells, but did not observe any change to MDCK^YBX1^ cell migration and invasion. As some EMT hallmarks were observed, but to a lesser magnitude than in 21D1 cells, it appears YBX1 may stimulate a partial EMT, or onset of the metastable EMT phenotype [36]. Previously, YBX1 expression in MCF10A cells did not cause EMT, however, expression in MCF10AT (Ras-transformed MCF10A) cells induced the EMT phenotype [26]. This suggests that YBX1 alone cannot complete EMT, but can cooperate with other EMT modulators such as TGF-β [29] to enhance oncogenic cell properties. Elevated expression of YBX1 is commonly observed in cancer tissues, including ~75% of human breast carcinomas [37]. Furthermore, knock-down of YBX1 in gastric cancer cell lines inhibits their migration [38], and substantially hinders tumour growth [27, 39, 40].

We investigated whether elevated expression of YBX1 alone could increase tumourigenicity of MDCK cells. Subcutaneous injection of parental MDCK cells did not form tumours. Strikingly, MDCK^YBX1^ cells established viable tumour xenografts. We were interested in exploring both the cellular and extracellular proteins that may contribute to the tumourigenicity of MDCK^YBX1^ cells. Given that we could not compare the tumour protein expression directly, we instead subjected the *in vitro* cells lines to proteomic profiling. We identified 57 cellular proteins that were significantly elevated in MDCK^YBX1^ cells, compared to MDCK cell lysates, and 11 which are mitochondrial constituents. Additionally, we were able to identify ACADSB, SLC25A1, IARS2, and OAT of these cellular proteins in the MDCK^YBX1^ cell xenografts, suggesting their involvement in tumorigenesis. It is tempting to speculate that these mitochondrial proteins may facilitate metabolic requirements needed for tumour formation. We next examined the molecular mechanisms associated with MDCK^YBX1^ cells establishing tumour xenografts.

We first investigated MDCK^YBX1^ cell propensity for anchorage-independent growth, given the elevation in *in vitro* colony forming assays. Anchorage-independent growth is a crucial step in the acquisition of EMT [41]. EMT transcription factors Snail1 and Twist had increased expression in MDCK^YBX1^ cells, and Snail1 is known to promote anoikis resistance by inhibiting caspase-3 activity and activating the pro-survival PI3K-Akt pathway [42, 43]. Additionally, DSG2 and BAX had reduced expression in MDCK^YBX1^ cells, and given BAX is a pro-apoptotic regulator, its decrease may contribute to overcoming anoikis, and the progression of anchorage-independent growth [44].

We next explored the involvement of YBX1 in promoting angiogenesis. MDCK^YBX1^ cell xenografts stained positive for CD31, indicating murine vascularization. We also identified abundant expression of proteins involved in angiogenesis (ANXA2, CA2, ATP5B and HSPB1) [45, 46] in MDCK^YBX1^ tumours. Given that a blood supply is critical for tumour growth, as well as dissemination [47], we hypothesized that elevated expression of YBX1 in MDCK cells may promote tumour angiogenesis. We reasoned that extracellular molecules may facilitate this process, and would be released from the tumour into the microenvironment. Encouragingly, secretome from MDCK^YBX1^ cells enhanced the *in vitro* migration of 2F-2B cells, compared to cells stimulated with MDCK secretome, suggesting the involvement of extracellular factors. To this end, we performed comparative antibody arrays and relative proteomic profiling of the secretome to identify the molecules which may enhance cell migration. A key finding was the elevated expression in MDCK^YBX1^ cell secretome of various proteases (ADAM9 and ADAM17) and several stimulatory/growth factors (TGF-β1, CSF-1, NGF, and VGF). Interestingly, these molecules have been implicated in cancer progression and angiogenesis [48].

ADAM proteases describe a family of proteins which typically contain an N-terminal prodomain, a metalloproteinase domain, a disintegrin, a cysteine-rich, a transmembrane, and cytoplasmic domain [49, 50]. ADAM9 has two isoforms, the second which lacks the C-terminal region of the protein (transmembrane and cytoplasmic domain), and is thus secreted [51]. Importantly, it is this secreted isoform that is known to be expressed in breast cancer cell lines and tumours, and promotes breast cancer cell migration and invasion [52]. ADAM9 is also known to cleave EGF to promote tumour growth and facilitate angiogenesis [53, 54]. In developing chicken embryos, ADAM17 has direct implications in promoting angiogenesis and facilitating blood vessel sprouting [55]. ADAM17 activity was shown to promote pericyte cell proliferation and enlarge radial microvessels [55]. With respect to tumorigenesis, ADAM17 is generally considered to activate EGF-related growth factors via proteolytic processing to stimulate cell proliferation and migration [56]. We anticipate that elevated levels of these ADAM proteases in the tumour microenvironment would have pleiotropic functions including degradation of endothelial basement membranes, removal of ECM for cell migration, and activation/release of growth factors and receptors.

Elevated expression of CSF-1 and TGF-β1 in the secretome of MDCK^YBX1^ cells could promote several aspects of carcinogenesis, as these molecules have been heavily implicated in cancer-related processes [57–59]. However, TGF-β1 which was the most significantly up-regulated factor on the array has been shown to induce angiogenesis *in vivo* [60–62]. Similarly, NGF promotes angiogenesis in breast cancer by increasing the invasion and cord formation of endothelial cells, and antibody-based neutralization inhibits this *in vivo* [63], leading NGF to be considered a candidate for therapeutic intervention [64]. Excitingly, NGF is known to induce VGF expression [65], and our results suggest they may be working cooperatively. Nonetheless, the precise role of VGF in angiogenesis and cancer progression remains less characterized. Our observations reinforce the complexity of the tumour microenvironment, as we identified the elevated expression of numerous growth/differentiation factors simultaneously. It is possible that these molecules collaborate to drive different elements of angiogenesis such as endothelial activation, proliferation, migration, and tissue infiltration.

In summary, our study revealed that elevated YBX1 expression induced a partial EMT in MDCK cells, causing cell scattering, relocalization of E-cadherin, and increased levels of EMT transcription factors. As YBX1 only induced a partial EMT pheonotype, clearly, additional modulators are required to complete EMT (such as those observed in Ras-transformed 21D1 cells). A salient finding of this study was the observation that in contrast to parental MDCK cells, MDCK^YBX1^ cells exhibit pronounced tumourigenicity both *in vitro* (anchorage-independent growth), and *in vivo* (established tumour xenografts). Proteomic profiling of MDCK^YBX1^ cell-derived secretome revealed significantly elevated expression of several proteases and extracellular factors involved in promoting angiogenesis and endothelial cell migration. Studies are underway to determine the molecules which directly promote EMT, tumorigenesis, and neovascularization, towards design of therapeutic strategies that may reverse EMT and limit cancer progression.

## MATERIAL AND METHODS

### Cell culture and YBX1 transfection

MDCK [66], MDCK^YBX1^, 21D1 [20, 21] and 2F-2B cells (ATCC) were maintained in Dulbecco's Modified Eagle's Medium (DMEM) with high-glucose (Invitrogen-GIBCO, Carlsbad, CA, USA) supplemented with 10% (v/v) Fetal Bovine Serum (FCS) (Invitrogen-GIBCO), 1% (v/v) Penicillin Streptomycin (Pen/Strep) (Life Technologies) and incubated at 37°C with 10% CO_2._ YBX1 was transfected (pcDNA3.1(+) vector, Life Technologies) into MDCK cells using X-tremeGENE9DNA (Roche). Transfected cells were selected using G418 geneticin (Life Technologies) 24 hr post-transfection.

### Cell growth assay

Cells were seeded (5 × 10^3^) and cultured for 24 hr, prior to commencement of assay. Every 24 hr, for a further 4 days, each cell line from a single well was washed with PBS, lifted with 0.1% (v/v) trypsin-versene, and counted with a haemocytometer. Cells in four fields of view were averaged, and assays repeated in triplicate.

### Cell lysate preparation

Cells (1 × 10^6^) were washed (ice cold PBS) and lysed on ice with SDS sample buffer (4% (w/v) SDS, 20% (v/v) glycerol, 0.01% (v/v) bromophenol blue, 0.125 M Tris-HCl, pH 6.8). Lysates were subjected to ultracentrifugation for 30 min at 4°C (386, 000 × *g*, TLA-100 rotor, Beckman Coulter), and soluble supernatants aspirated for downstream use, or frozen at −80°C.

### Microscopy

#### Phase contrast

Cells (1 × 10^6^) were washed with DMEM and imaged on an inverted Nikon Eclipse TE300 microscope equipped with a 10x objective (Nikon Plan Fluor) in phase-contrast mode using an attached 12.6 mp digital camera (Nikon DXM1200C). Images obtained from MDCK, MDCK^YBX1^ and 21D1 cells (10 independent fields of view) were processed with Nikon Elements Imaging Software (v.3.00, SP$ (Build 502)).

#### Confocal

Cells were fixed in 4% (v/v) formaldehyde in PBS, semi-permeablised (0.2% (v/v) Triton X-100 in PBS), washed with wash buffer (0.1% (w/v) BSA and 0.1% (v/v) Tween-20 in PBS) and blocked (5% (v/v) goat serum, 5% (w/v) BSA, 0.1% (w/v) cold fish gelatin, 0.05% (v/v) Tween-20, 0.05% (w/v) sodium azide, 0.01 M PBS, pH 7.2) for 2 h. Cells were incubated with primary antibodies [mouse anti-E-cadherin (BD Transduction Laboratories; 1:500), rabbit anti-YBX1 (Abcam; 1:500), mouse anti-Vimentin (Merck Millipore; 1:200)] for 1 hr, washed in wash buffer and incubated at RT with either Alexa Fluor 488-conjugated goat anti-mouse IgG or AlexaFluor 546-conjugated goat anti-rabbit IgG (Life Technologies). Cells were incubated with To-pro-3 (1:1000 in wash buffer, Life technologies), washed, and imaged on a Zeiss LSM 780 confocal microscope with 100x magnification.

### Protein quantification and immunoblotting

Protein quantification was performed using 1D-SDS-PAGE / SYPRO^®^ Ruby protein staining densitometry, as previously described [23]. For immunoblotting (10 μg), membranes were probed with primary antibodies [mouse anti-E-cadherin (BD Transduction Laboratories; 1:1000), mouse anti-H-Ras (Santa Cruz Biotechnology; 1:1000), rabbit anti-YBX1 (Abcam; 1:1000), mouse anti-Vimentin (Merck Millipore; 1:1000), mouse anti-β-actin (Cell Signalling Technologies; 1:2000), mouse anti-Claudin1 (Santa Cruz Biotechnology; 1:1000), rabbit anti-Snail1 (Abcam; 1:1000), rabbit anti-Twist (Santa Cruz Biotechnology; 1:1000), or mouse anti-GAPDH (Life technologies; 1:12, 000) for 1 hr at RT in TTBS (50 mM Tris, 150 mM NaCl, 0.05% (v/v) Tween 20) followed by incubation with either IRDye 800 goat anti-mouse IgG or IRDye 700 goat anti-rabbit IgG (1:15000, LI-COR Biosciences) for 1 hr at RT in TTBS. Immunoblots were imaged using the Odyssey Infrared Imaging System, (v3.0, LI-COR Biosciences, Nebraska USA).

### Nuclear and cytoplasmic extraction

Cell-derived nuclear and cytoplasmic extracts were obtained as previously described [67]. Nuclear and cytoplasmic extracts (10 μg), were subjected to immunoblotting.

### Secretome preparation

Secretome purification was performed as previously described [68], with modifications. Briefly, MDCK, MDCK^YBX1^ and 21D1 cells were grown to 80% confluence in DMEM containing 10% FCS, subjected to 3 × washes with DMEM (0% FCS) and cultured in DMEM for 24 h. Cell conditioned medium was collected and centrifuged at 500 × *g* for 5 min, 2000 × *g* for 10 min to sediment floating cells and remove cell debris. Conditioned medium was concentrated by centrifugal ultrafiltration (3K NMWL Ultra-15, Merck-Millipore) at 3000 × *g* to obtain secretome samples.

### Wound healing, invasion, and colony forming assay

The wound healing/scratch cell migration assays were performed as described previously [69]. Transwell invasion assays were performed as previously described [70]. Soft agar growth assays were performed as previously described [71]. All assays were performed in triplicate.

### Transwell migration assay

For 2F-2B endothelial cell migration assays, 5 × 10^4^ cells in suspension were stimulated (vehicle control DMEM) with 30 μg secretome derived from MDCK, MDCK^YBX1^ or 21D1 cells for 2 hr at 37°C. Cells were pelleted at 500 × *g* for 5 mins, and resuspended in 100 μL of DMEM. Cells were overlaid onto Transwell^®^ polycarbonate membrane cell culture inserts (8.0 μm pore size, Corning), and inserts placed into 24-well companion plates. The bottom chamber contained vehicle control DMEM + 5% FCS, and was supplemented with 30 μg MDCK, MDCK^YBX1^, or 21D1 secretome. Cell migration through the transwell was performed at 37°C for 24 hr. Inserts were removed, and cells fixed (4% (v/v) formaldehyde, 10 min) and nuclei stained with DAPI. Non-migrating cells were removed from the upper side of the inserts using cotton swabs. Migrating cells were imaged using an inverted Nikon Eclipse TE300 microscope equipped with an attached 12.6 mp digital camera (Nikon DXM1200C) (*n* = 3; average ± SEM, **P < 0.01).

### Angiogenesis antibody array

Cell-derived secretomes (150 μg), were collected (as above) and applied to angiogenesis antibody arrays, according to manufacturer's instructions (ARY007, R&D Systems). Assays were performed in triplicate.

### Tumour xenografts

All experiments were performed according to guidelines of La Trobe University Ethics committee. For xenograft assays, cells (1 × 10^6^ cells/site) were injected subcutaneously into NOD/SCID male mice (*n* = 8) in both inguinal regions. Primary tumour volumes were calculated according to V= (small diameter)^2^ × (larger diameter) × 0.5 [72]. Animals were sacrificed 5 weeks post-injection, tumours excised and subjected to either protein extraction or fixed in 4% paraformaldehyde, and paraffin embedded for histological examination. For protein extraction, lysis buffer (5 mL of (4% (w/v) SDS, 20% (v/v) glycerol and 0.01% (v/v) bromophenol blue, 0.125 M Tris-HCl, pH 6.8)) with protease inhibitor cocktail (Complete, EDTA-free protease inhibitor cocktail, Roche) and 1 mM DTT was combined with homogenised tumours and sonicated for 180 sec. Homogenates were incubated at 95°C for 20 min and 60°C for 2 hr. After centrifugation at 25,000 *g* for 30 min, each supernatant was subjected to quantification. Fresh sections (20 μm) were prepared using a vibratome (Leica VT 1000S) and subjected to immunostaining as described [73]. Incubations with primary antibody (rabbit anti-CD31 (Abcam, 1:50)) performed overnight at 4°C and detection by direct immunofluorescence with AlexaFluor 546-conjugated goat anti-rabbit IgG (Life Technologies). Nuclei were stained with To-pro-3 (1:1000) and imaged using Zeiss LSM 780 confocal microscope (63x magnification).

### Proteomic analysis

Proteomic experiments were performed in duplicate.

#### Cell lysates and tumour xenografts

Cell lysates (10 μg) and tumour xenograft extracts (10 μg) were lysed in SDS sample buffer, and proteins separated by SDS-PAGE and visualized by Imperial™ Protein Stain (Thermo Fisher Scientific). Individual samples were excised and destained (50 mM ammonium bicarbonate/acetonitrile), reduced (10 mM DTT (Calbiochem) for 30 min), alkylated (50 mM iodoacetic acid (Fluka) for 30 min) and trypsinized (0.2 μg trypsin (Promega Sequencing Grade) for 16 hr at 37°C), as described [74].

#### Secretome

Samples (50 μg) were lysed with ProteaseMAX™ Surfactant (0.15% w/v) (Promega) and 8 M urea. Proteins were reduced and alkylated as above, and in-solution digestion performed (1 μg trypsin), as described [75].

For all samples, peptides were desalted using reverse-phase C18 StageTips [76], and eluted in 85% (v/v) acetonitrile (ACN) in 0.5% (v/v) formic acid (FA). Peptides were lyophilised in a SpeedVac and acidified with buffer containing 0.1% FA, 2% ACN. A nanoflow UPLC instrument (Ultimate 3000 RSLCnano, Thermo Fisher Scientific) was coupled on-line to an Orbitrap Elite mass spectrometer (Thermo Fisher Scientific) with a nanoelectrospray ion source (Thermo Fisher Scientific). For cell lysates and tumour xenograft samples, ~2 μg peptides were loaded (Acclaim PepMap100 C18 5 μm 100 Å, Thermo Fisher Scientific) and separated (Vydac MS C18-RP column, 25 cm, 75 μm inner diameter, 3 μm 300 Å, Grace, Hesperia, CA) with a 120-min linear gradient from 0–100% (v/v) phase B (0.1% (v/v) FA in 80% (v/v) ACN) at a flow rate of 250 nL/min. For secreted samples, ~10 μg peptides were loaded (Acclaim PepMap100 C18 5 μm 100 Å, Thermo Fisher Scientific) and separated (PepMapRSLC C18, 50 cm, 75 μm inner diameter, 2 μm 100 Å, Thermo Fisher Scientific) with a 300-min linear gradient from 0–40% (v/v) phase B (0.1% (v/v) FA in 80% (v/v) ACN), at a flow rate of 250 nL/min.

The mass spectrometer was operated in data-dependent mode where the top 20 most abundant precursor ions in the survey scan (300–2500 Th) were selected for MS/MS fragmentation. Survey scans were acquired at a resolution of 120, 000 at m/z 400. Unassigned precursor ion charge states and singly charged species were rejected, and peptide match disabled. The isolation window was set to 3 Th and selected precursors fragmented by CID with normalized collision energies of 25. Maximum ion injection times for the survey scan and MS/MS scans were 20 ms and 60 ms, respectively, and ion target values were set to 3E6 and 1E6, respectively. Dynamic exclusion was activated for 90 sec.

### Database searching and protein identification

Raw data was processed using MaxQuant [77] (v1.1.1.25) and searched with Andromeda using either Canine-only (UniProt #28698 entries) or Canine-Mouse (UniProt #105098 entries) sequence databases (Oct-2014). Data was searched with a parent tolerance of 10 ppm, fragment tolerance of 0.5 Da and minimum peptide length 7. FDR was 1% at the peptide and protein levels, and data examined with label-free quantitation (LFQ) [78]. LFQ intensities for all unique and razor peptides were included, with zero intensity values replaced with a constant value of 1 to calculate fold change ratios. LFQ intensity values were averaged and fold change ratios calculated. Contaminants, and reverse database identifications were excluded from further data analysis. Proteins commonly identified in both replicate experiments were used to compare against other cell samples.

### Statistical analysis

Student's *t*-tests (GraphPad v5.0) were calculated, with *p < 0.05 and **p < 0.01 considered statistically significant.

## SUPPLEMENTARY FIGURES AND TABLES















## ABBREVIATIONS

21D1: Ras-transformed MDCK cells
ADAM9: a disintegrin and metalloproteinase domain-containing protein 9
DMEM: Dulbecco's Modified Eagle's Medium
EMT: epithelial-mesenchymal transition
FCS: fetal calf serum
MDCK: Madin-Darby canine kidney
MMP1: matrix metalloproteinase 1
NMWL: nominal molecular weight limit
YBX1/YB-1: nuclease-sensitive element-binding protein 1/Y-box binding protein 1
