# Supplementary material for: YBX1/YB-1 induces partial EMT and tumourigenicity through secretion of angiogenic factors into the extracellular microenvironment
Source: Oncotarget. 2015 Apr 23;6(15):13718–30. doi: 10.18632/oncotarget.3764 (PMC4537044; doi:10.18632/oncotarget.3764)
Supplement: Supplementary file 1 [file oncotarget-06-13718-s001.pdf]

SUPPLEMENTARY FIGURES AND TABLES

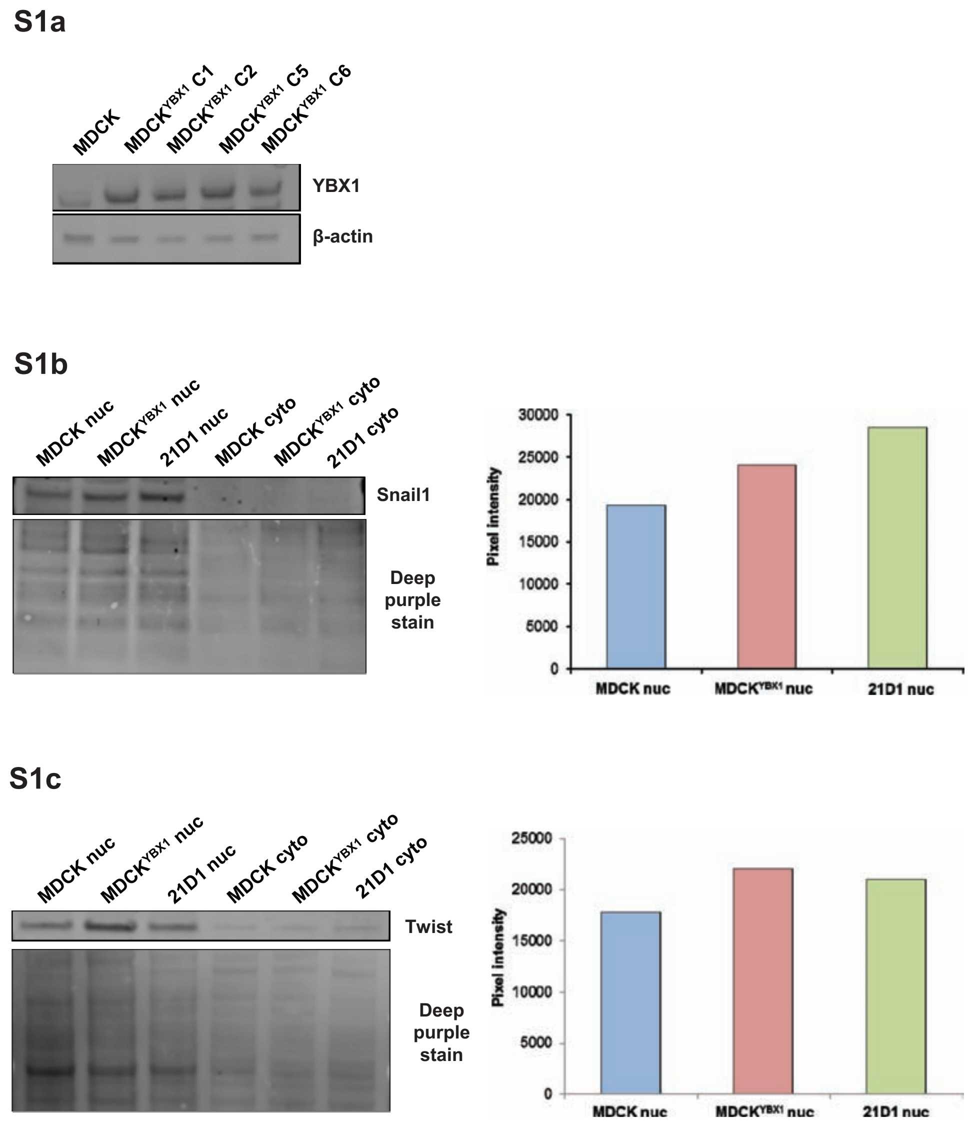

Supplementary Figure S1: YBX1 overexpression in MDCK cells. a. Immuno-blotting analysis of YBX1 in MDCK cells. Cells were stably transfected with the full-length sequence of YBX1 cDNA and selected as single clones (C1-2, 5-6). Immuno-blotting (10 µg) revealed elevated YBX1 expression in clone 5 (C5, MDCK^{YBX1}). **b-c.** Immuno-blotting analysis of Snail1 and Twist expression in nuclear and cytosolic extracts. Densitometry analysis revealed an increase in expression of Snai1 and Twist in MDCK^{YBX1} in comparison to MDCK nuclear extracts. Target intensity was normalized in relation to total protein intensity (deep purple stain).

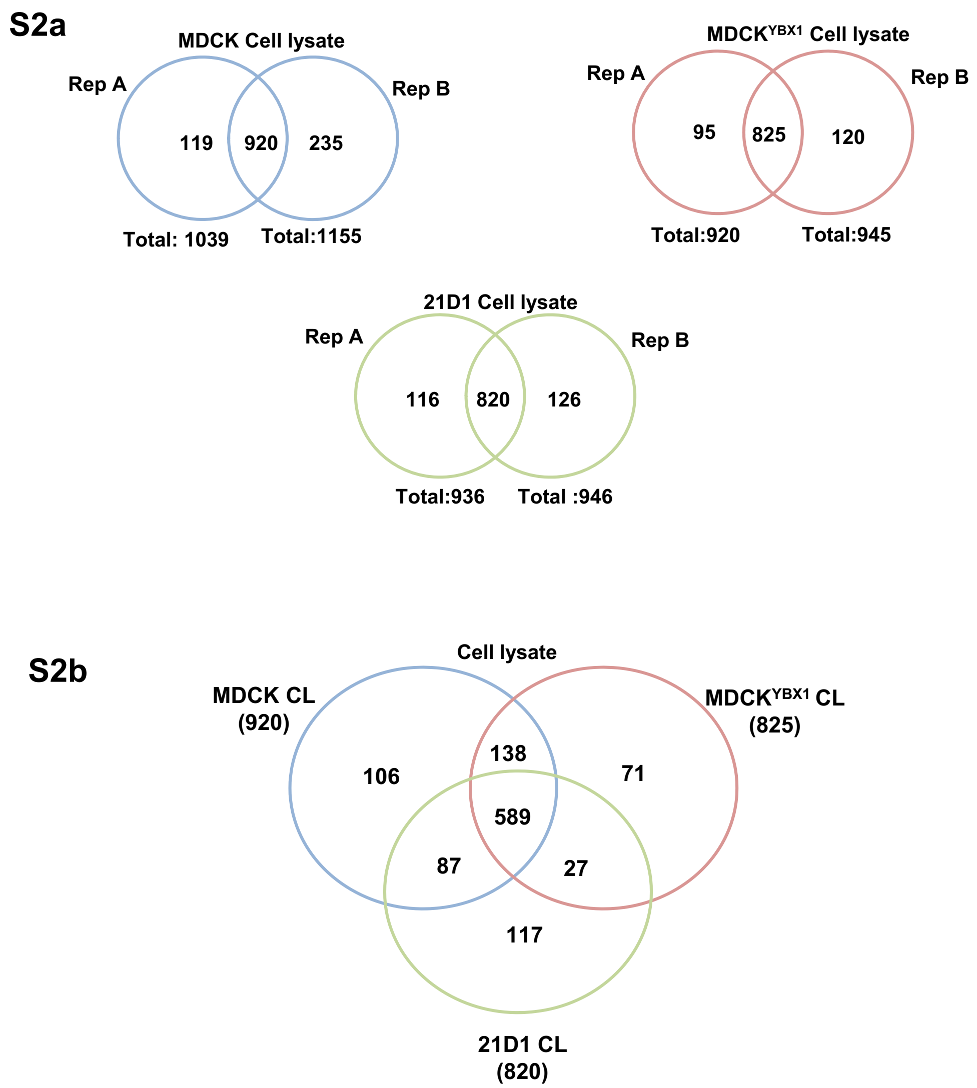

Supplementary Figure S2: Identification of MDCK, MDCK^{YBX1} and 21D1 cellular proteins. a. Reproducibility of protein identification across individual replicates. **b.** Comparison of proteins identified in both replicates, between cell lines.

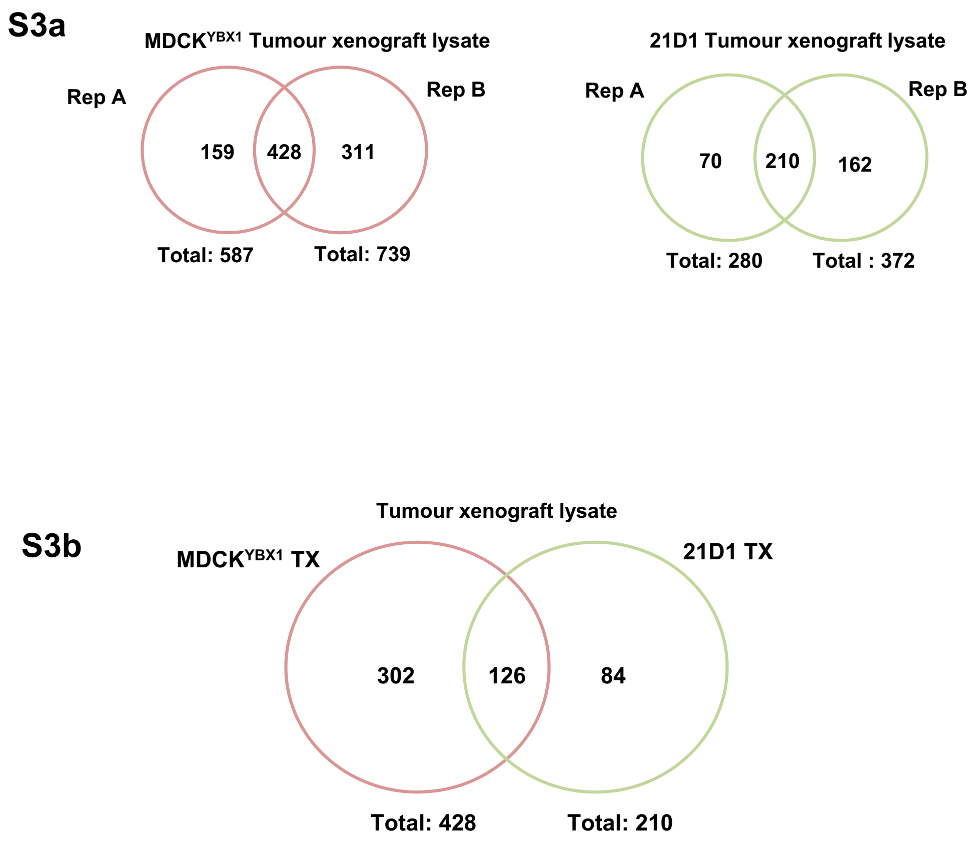

Supplementary Figure S3: Identification of MDCK^{YBX1} and 21D1 tumour xenograft proteins. a. Reproducibility of protein identification across individual replicates. **b.** Comparison of proteins identified in both replicates, between cell lines.

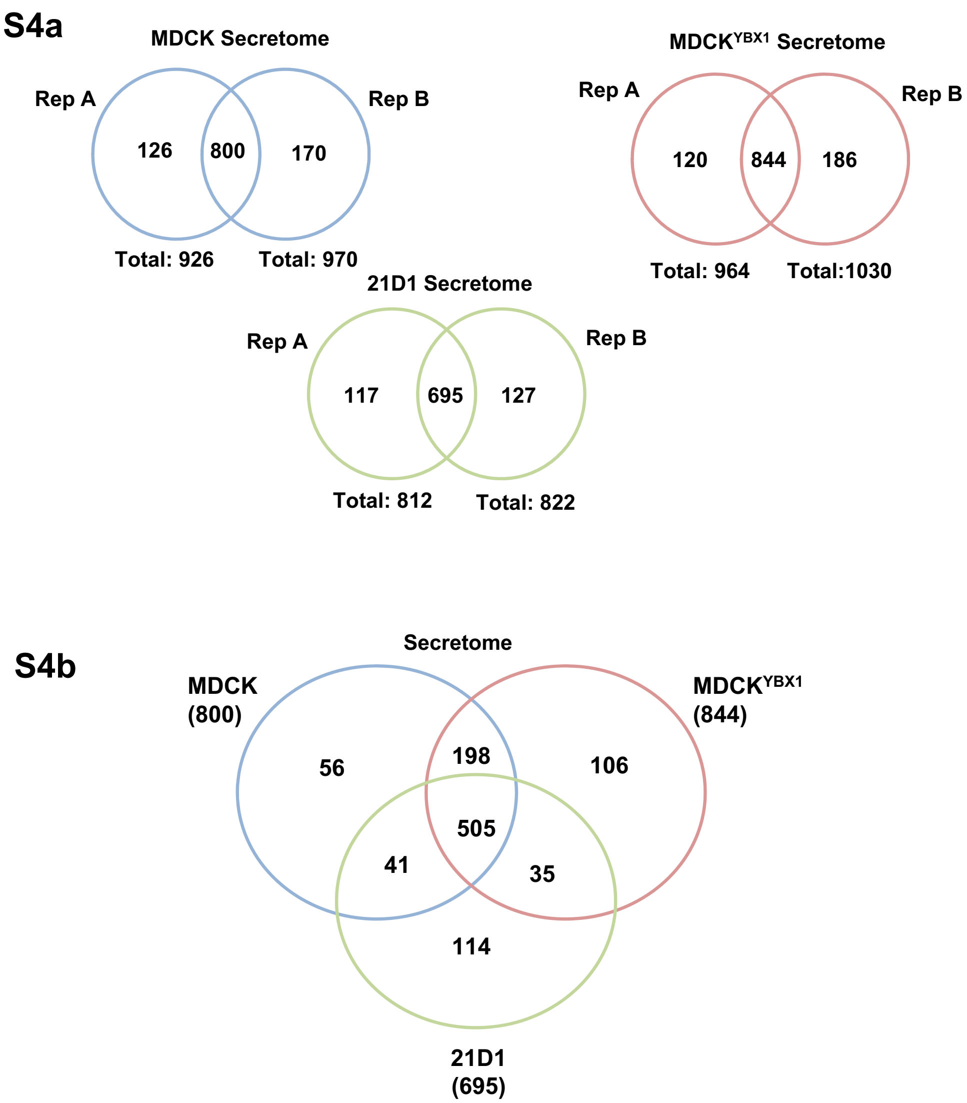

Supplementary Figure S4: Identification of MDCK, MDCK^{YBX1} and 21D1 secretome proteins. a. Reproducibility of protein identification across individual replicates. **b.** Comparison of proteins identified in both replicates, between cell lines.

Supplementary Table S1. Proteins identified in MDCK, MDCK^{YBX1}and 21D1 CL. LFQ intensities and ratios (fold-change) of proteins in replicates of MDCK, MDCK^{YBX1} and 21D1 cell lysates.

Supplementary Table S2. Proteins significantly enriched in MDCK^{YBX1} in comparison to MDCK CL. Ratios of LFQ intensities (fold-change) of proteins enriched in MDCK^{YBX1} in comparison to MDCK **cell** lysates.

Supplementary Table S3. Proteins identified in MDCK^{YBX1} and 21D1 TX. LFQ intensities and ratios of proteins in replicates of MDCK^{YBX1} and 21D1 tumor xenograft lysates.

Supplementary Table S4. Proteins identified in MDCK, MDCK^{YBX1} and 21D1 Secretome. LFQ intensities and ratios (fold-change) of proteins in replicates of MDCK, MDCK^{YBX1} and 21D1 secretome.

Supplementary Table S5. Proteins significantly enriched in MDCK^{YBX1} in comparison to MDCK secretome. Ratios of LFQ intensities (fold-change) of proteins enriched in MDCK^{YBX1} in comparison to MDCK Sec.

Supplementary Table S6. Proteins significantly enriched in 21D1 in comparison to MDCK secretome. Ratios of LFQ intensities (fold-change) of proteins enriched in 21D1 in comparison to MDCK Sec.
